# A Clear View of Mycobacterial Infection

**DOI:** 10.1371/journal.pbio.0020410

**Published:** 2004-10-26

Fighting an infection might seem to be a battle between David and Goliath, given the relative sizes of bacterial infectious agents and the animals they infect. But on closer examination it is more often a chess match between two skilled opponents who have the uncanny ability to anticipate each other's moves. Mycobacterium tuberculosis causes tuberculosis (TB) in people, and related species that infect other animals are used as model systems for the study of TB. Much progress has been made in identifying the armaments (or virulence factors) of the bacteria. But the interplay, or chess match, between the bacterium and the animal it infects is much less clear. One of the host's first moves against the mycobacterium is the formation of a granuloma. Granulomas are tightly aggregated structures that consist of macrophages—one of the first lines of defense of the immune system—within which the infecting bacterium grows. Besides these and related cells that are present at the site of infection, additional macrophages and other immune cells are recruited in the formation of the granuloma. Although granulomas are required for the elimination of the infection, Lalita Ramakrishnan and colleagues have now shown that the bacteria have a game plan of their own.

**Figure:**
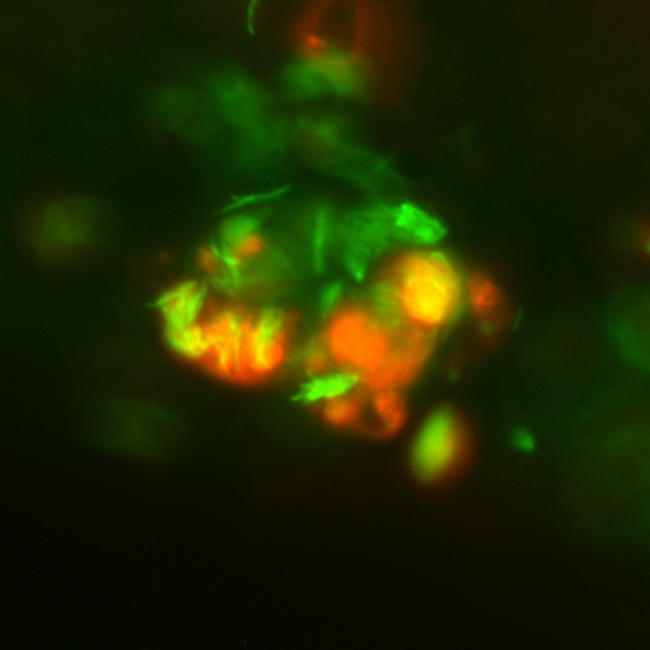
Mycobacteria co-opt granulomas for their growth and spread

One problem in understanding the interaction between the mycobacterium and the host has been that it occurs deep in the lung of the infected animal, which makes it difficult to analyze how each of the animal or bacterial factors affect the strategic interplay between the host and pathogen. To overcome this limitation, Ramakrishnan and colleagues used zebrafish embryos, which are transparent and can be infected by a relative of the TB pathogen, M. marinum. This enables the researchers to watch cells as they are recruited into the granuloma.

Some of the virulence factors of mycobacteria are encoded in an area of the genome called the RD1 locus. In a mouse model, a strain of the bacteria missing RD1 causes far less pathology than a strain with the full complement of genes. The RD1 locus is also absent in the bacterial strain M. bovis that is used as an attenuated TB vaccine. But the precise role of RD1 in infection remains obscure.

By visualizing in zebrafish infections of a virulent strain of M. marinum and a strain with an RD1 deletion, Ramakrishnan and colleagues have observed that RD1 is actually required for granuloma formation but isn't needed for the bacteria to infect macrophages. What's more, macrophages that are infected with mycobacteria that contain RD1 produce a signal that further recruits macrophages to granulomas. This might seem an odd virulence strategy, as macrophages are required for mycobacterial elimination. But in this ongoing chess match, the virulent mycobacterium exploits the host's defense—granuloma formation—by providing additional macrophages for the bacteria to infect.

The end game of the chess match remains unclear. While granulomas are required for protection against mycobacteria, they are not completely effective. Thus, these bacteria have developed a strategy to recruit the normally defensive cells of the host to their advantage, but it remains to be shown what tips the balance between the macrophages' ability to clear the infection and their unwitting participation in the development of TB.

